# Deep learning assisted diagnosis system: improving the diagnostic accuracy of distal radius fractures

**DOI:** 10.3389/fmed.2023.1224489

**Published:** 2023-08-17

**Authors:** Jiayao Zhang, Zhimin Li, Heng Lin, Mingdi Xue, Honglin Wang, Ying Fang, Songxiang Liu, Tongtong Huo, Hong Zhou, Jiaming Yang, Yi Xie, Mao Xie, Lin Lu, Pengran Liu, Zhewei Ye

**Affiliations:** ^1^Department of Orthopedics, Union Hospital, Tongji Medical College, Huazhong University of Science and Technology, Wuhan, China; ^2^Intelligent Medical Laboratory, Union Hospital, Tongji Medical College, Huazhong University of Science and Technology, Wuhan, China; ^3^School of Artificial Intelligence and Automation, Huazhong University of Science and Technology, Wuhan, China; ^4^Department of Orthopedics, Nanzhang People’s Hospital, Nanzhang, China; ^5^Department of Orthopedics, Renmin Hospital of Wuhan University, Wuhan, China

**Keywords:** artificial intelligence, deep learning, distal radius fractures, computer-assisted diagnosis, elderly population groups

## Abstract

**Objectives:**

To explore an intelligent detection technology based on deep learning algorithms to assist the clinical diagnosis of distal radius fractures (DRFs), and further compare it with human performance to verify the feasibility of this method.

**Methods:**

A total of 3,240 patients (fracture: *n* = 1,620, normal: *n* = 1,620) were included in this study, with a total of 3,276 wrist joint anteroposterior (AP) X-ray films (1,639 fractured, 1,637 normal) and 3,260 wrist joint lateral X-ray films (1,623 fractured, 1,637 normal). We divided the patients into training set, validation set and test set in a ratio of 7:1.5:1.5. The deep learning models were developed using the data from the training and validation sets, and then their effectiveness were evaluated using the data from the test set. Evaluate the diagnostic performance of deep learning models using receiver operating characteristic (ROC) curves and area under the curve (AUC), accuracy, sensitivity, and specificity, and compare them with medical professionals.

**Results:**

The deep learning ensemble model had excellent accuracy (97.03%), sensitivity (95.70%), and specificity (98.37%) in detecting DRFs. Among them, the accuracy of the AP view was 97.75%, the sensitivity 97.13%, and the specificity 98.37%; the accuracy of the lateral view was 96.32%, the sensitivity 94.26%, and the specificity 98.37%. When the wrist joint is counted, the accuracy was 97.55%, the sensitivity 98.36%, and the specificity 96.73%. In terms of these variables, the performance of the ensemble model is superior to that of both the orthopedic attending physician group and the radiology attending physician group.

**Conclusion:**

This deep learning ensemble model has excellent performance in detecting DRFs on plain X-ray films. Using this artificial intelligence model as a second expert to assist clinical diagnosis is expected to improve the accuracy of diagnosing DRFs and enhance clinical work efficiency.

## Introduction

Distal radius fracture (DRF) is one of the most common fractures, accounting for about 20% of all fractures and about 17–25% of emergency cases (1, 2). DRFs are more common in middle-aged and elderly patients with osteoporosis, typically resulting from low-energy injuries like falls, while young patients are mostly caused by high-energy injuries (3). With the increasing severity of aging, the incidence of the disease also shows an increasing trend year by year (4).

As a fast and inexpensive imaging tool, X-ray examination is the preferred method for evaluating wrist injuries (5). However, in emergency departments or outpatient clinics, non-orthopedic surgeons or young radiologists are often the first doctors to evaluate X-rays and need to make urgent decisions. Unfortunately, misdiagnosis (especially missed fractures) is common in these scenarios due to factors such as heavy workloads, fatigue, and lack of experience (6).

If patients’ fractures are not diagnosed in a timely manner, it may lead to delayed treatment, malunion and osteoarthritis, which will seriously affect their functional recovery, reduce their ability to live independently, and lower their quality of life (7). Especially for elderly patients, due to poor physical fitness and decreased body tissue function, if fractures do not get treated in time, the prognosis is more likely to be adversely affected (8). Therefore, accurate and efficient assistance technology for automated fracture detection has become a focus of attention.

Deep learning is a machine learning method, which realizes automatic feature extraction and expression by constructing a multi-layer neural network, so as to complete tasks such as data classification or regression (9, 10). Compared with traditional machine learning methods, deep learning does not require manual selection of features. Instead, it automatically learns features through neural networks, which greatly reduces the difficulty and complexity of feature engineering (11, 12). In recent years, with the progress in various aspects such as big data, high-performance computing, and algorithm optimization, deep learning has become a leading machine learning technology.

Image segmentation, object detection, and task classification based on deep learning technology have been successfully applied in the field of medical images, bringing new opportunities for the establishment of computer-aided medical imaging diagnosis systems. Deep learning techniques have been widely used in image analysis for a variety of diseases, including the detection of skin cancer, diabetic retinopathy, abnormal thyroid tissue abnormalities, and lung nodules (13–16). In recent years, deep learning has also been successfully applied to the identification and severity assessment of bone and joint lesions, such as knee joint lesions, osteoarthritis, and spinal degenerative lesions. In addition, some deep learning-based models have been used to assess bone age (17). Recently, more and more studies have shown that artificial intelligence models based on deep learning have great potential in fracture recognition, classification, segmentation, and visual interpretation (18–20). These model can significantly improve the accuracy of diagnosis, treatment, and prognosis evaluation, indicating its enormous potential applications in fractures.

In this study, we constructed a deep learning model to diagnose distal radius fractures using wrist AP and lateral X-ray images. (1) To train and evaluate the performance of DRF diagnosis using the first-stage model Faster R-CNN, the two-stage model RetinaNet, and the multi-stage model Cascade RCNN. (2) To build an expert-assisted system based on deep learning algorithm ensemble model through algorithm fusion to further improve the diagnostic accuracy of DRF and reduce misdiagnosis and missed diagnosis. (3) To compare the difference in diagnostic performance between the deep learning integrated model and clinical doctors. The results of this study further confirm the feasibility of deep learning-based assisted reading technology in clinical diagnosis. This technology is expected to provide a new, accurate and efficient aid for the clinical diagnosis of DRF.

## Methods

### Patients

This study was approved by our institutional ethics committee (IRB No.0840), and the requirement for informed consent was waived due to the retrospective nature of the study and negligible risks. All methods of this study were conducted in accordance with the Declaration of Helsinki.

**Figure 1:**
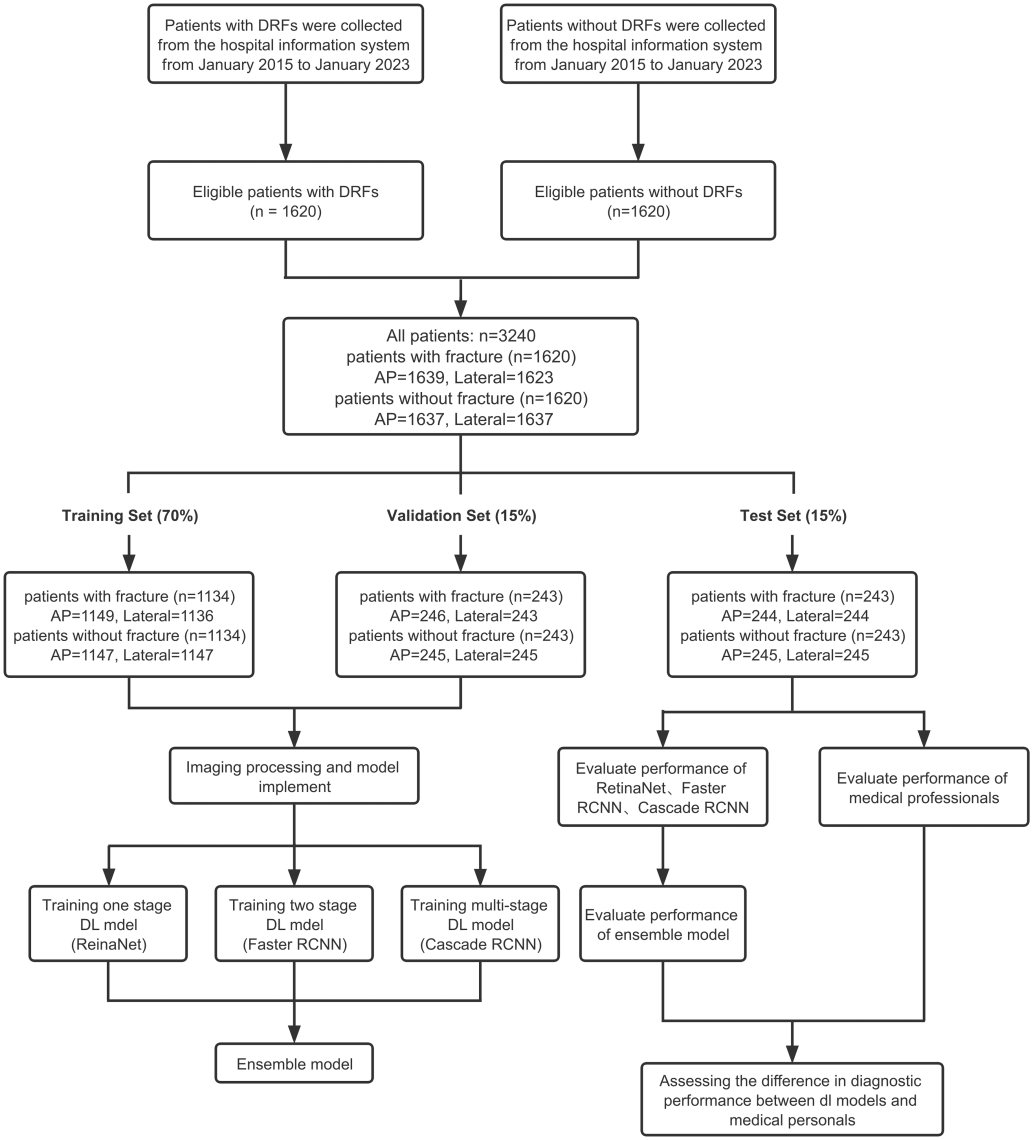
Flowchart of the entire research process.

Eligible patients were screened from the database of Union Hospital Affiliated to Tongji Medical College of Huazhong University of Science and Technology. Ultimately, 1,620 patients with DRFs who received treatment at the hospital from January 2014 to January 2023 were included. The inclusion criteria for patients are: (1) age over 18 years old; (2) diagnosed with DRF; (3) received X-ray examination. The exclusion criteria for patients include: (1) age under 18 years old; (2) old fractures, pathological fractures, and re-fractures after internal fixation; (3) foreign objects, such as plaster, jewelry, and clothing, that affect the final image judgment. Bilateral distal radius fractures were not our exclusion criteria. Furthermore, 1,620 patients without fractures whose diagnosis was sprain or carpal tunnel syndrome were included. Finally, all patients included in the study were divided into training set, validation set and test set according to the ratio of 7:1.5:1.5. The detailed inclusion process is shown in Figure 1.

### Diagnosis and annotation

The diagnosis of DRFs were mainly based on patients’ anteroposterior (AP) and lateral radiographs of wrist joint and combined with patients’ medical history. In some cases, computerized tomography (CT) were also used for a more comprehensive analysis. Two chief physicians, one from the orthopedics department and the other from the radiology department, with over 15 years of experience each, collaborated to make the diagnosis. In the case of disagreement, another chief orthopedic expert with 20 years of clinical experience would discuss with the two physicians and reached a final conclusion.

All obtained wrist joint radiographic images were saved in high-quality Joint Photographic Experts Group (JPEG) format. And, in order to protect the privacy of patients, all personal information (including name, gender, age and identity) on X-rays were hidden in the final obtained image.

**Figure 2:**
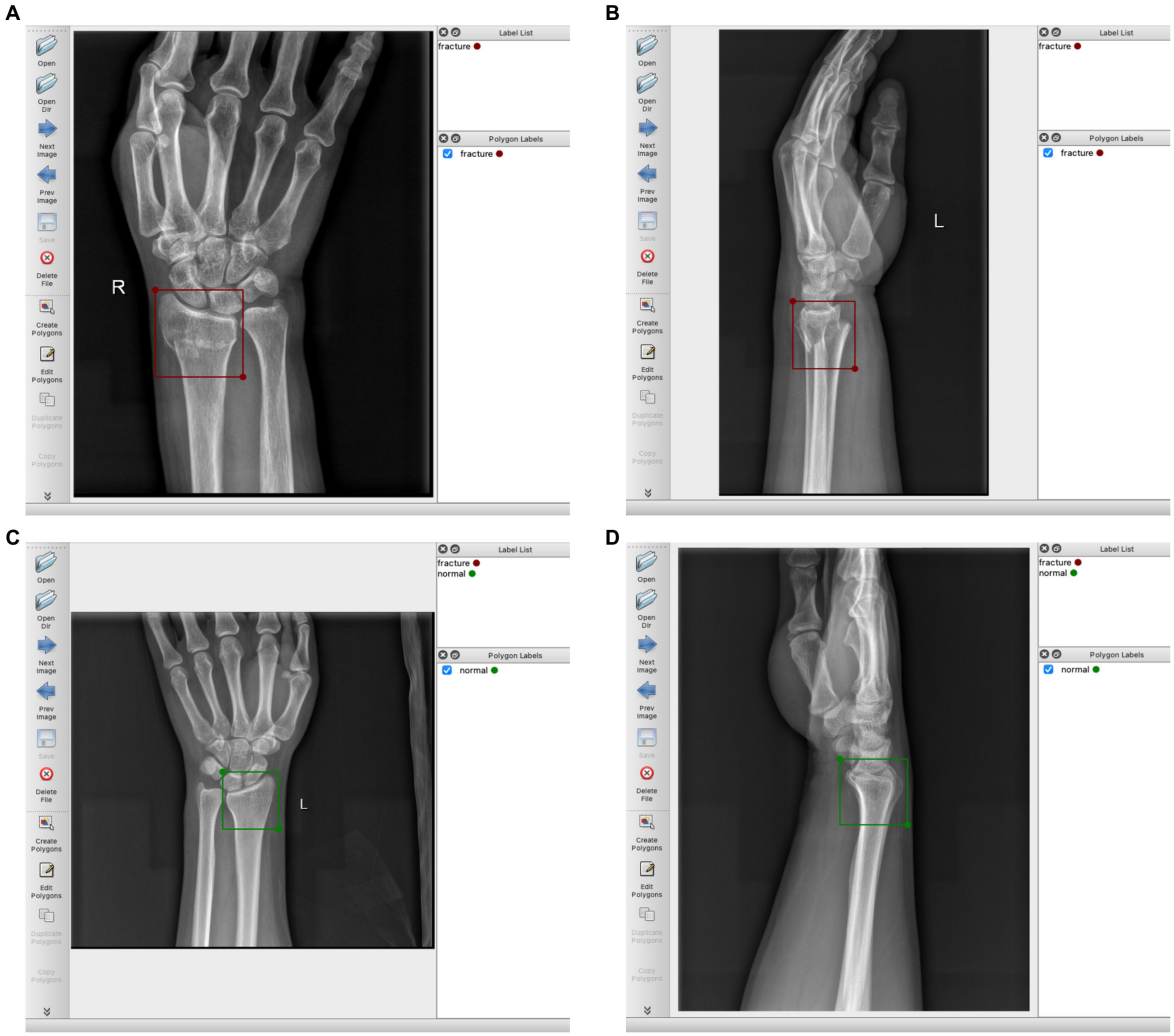
**(A)** Schematic diagram of labeling DRF AP radiograph, labeled as “fracture”; **(B)** DRF lateral radiograph, labeled as “fracture”; **(C)** Normal AP radiograph, labeled as “normal”; **(D)** Normal lateral radiograph, labeled as “normal”.

The region of the distal radius was drawn as a region of interest (RoI) using the rectangle tool, and was manually labeled according to the final imaging diagnosis (labels were divided into two categories: fracture and normal). We use the Labelme software package^1^ for manual labeling. Figure 2 provides a detailed illustration of our labeling process.

### Date set

Finally, we extracted a total of 3,276 images of the distal radius X-ray AP positions (1,639 fractures and 1,637 normal) and 3,260 images of the distal radius X-ray lateral positions (1,623 fractures and 1,637 normal) from the Picture Archiving and Communication System (PACS) at Union Hospital, covering a period from January 2014 to January 2023. A total of 16 lateral fracture radiographs were excluded because these images were taken in non-standard lateral position, possibly due to severe wrist pain that prevented standard positioning. These images were used for training, validation, and testing. There are a total of 2,296 AP view images in the training set (1,149 with fractures, 1,147 normal) and 2,283 lateral view images (1,136 with fractures, 1,147 normal). The validation set contains a total of 491 AP view images (246 with fractures, 245 normal) and 488 lateral view images (243 with fractures, 245 normal). The test set contains a total of 489 AP view images (244 with fractures, 245 normal) and 489 lateral view images (244 with fractures, 245 normal). The detailed division process is shown in Figure 1.

### Image processing

To improve the performance of object detection, we need to preprocess the data. To be more specific, we scaled the input image to have a short edge of 800 pixels and adjust the size of the long edge proportionally. At the same time, each image has a 50% chance of being horizontally flipped, increasing the richness of the images. In addition, we normalize the images and improve model training stability by converting the original images into a standard format through a series of transformations. The specific normalization parameters used are as follows: mean = [123.675, 116.28, 103.53] and std = [58.395, 57.12, 57.375].

### Development of deep learning ensemble model

**Figure 3:**
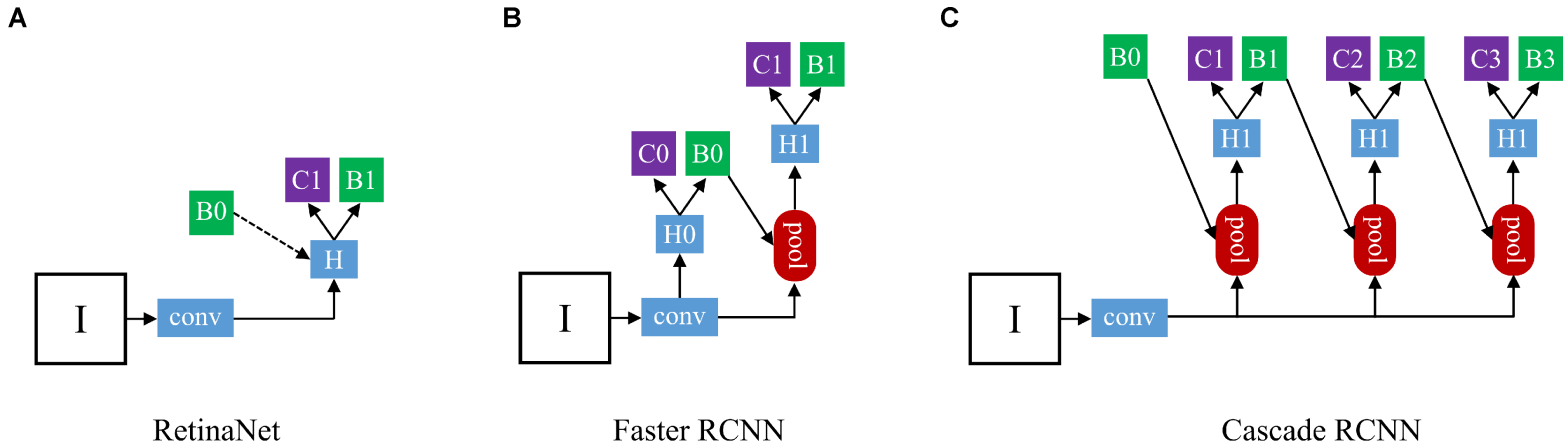
The architecture of three different types detection frameworks: **(A)** The structure of RetinaNet; **(B)** The structure of Faster RCNN; **(C)** The structure of Cascade RCNN. “I” is input image, “conv” backbone convolutions, “pool” region-wise feature extraction, “H” network head, “B” bounding box, and “C” classification. In RetinaNet, “B0” is proposals in all architecutes. In Faster RCNN and Cascade RCNN, “B0” pre-defined anchor box.

We chose three different types of deep learning models (Figure 3): one-stage RetinaNet (21), two-stage Faster RCNN (22), and multi-stage Cascade RCNN (23). As a first-stage algorithm, RetinaNet directly classifies and regresses the entire image to generate object detection results, with features such as fast speed and low computational complexity. Besides, RetinaNet’s design also incorporates Feature Pyramid Network (FPN), which can effectively handle objects of different scales, further improving detection accuracy and robustness (24). Faster R-CNN’s workflow is mainly divided into two stages: regional recommendation and target classification. In the Region Proposal phase, a new neural Network structure called Region Proposal Network (RPN) is introduced, which generates candidate regions on the input image by sliding windows. In the target classification phase, each candidate box is converted to a fixed-size feature graph by RoI Pooling the RPN-generated candidate boxes, and then entered into a full-connection layer classifier for target classification (25). Cascade R-CNN is a multi-stage model which includes three stages: candidate box generation, candidate box classification, and candidate box regression. In the first stage, Selective Search or other bounding box generation algorithms are used to generate a large number of candidate boxes. In the second stage, a cascade classifier method is adopted to cascade a series of classifiers together, with each classifier being stricter than the previous one, for further filtering of candidate boxes and selecting more accurate positive samples. In the third stage, a regression network is used to fine-tune the filtered candidate boxes to further improve detection accuracy (26).

**Figure 4:**
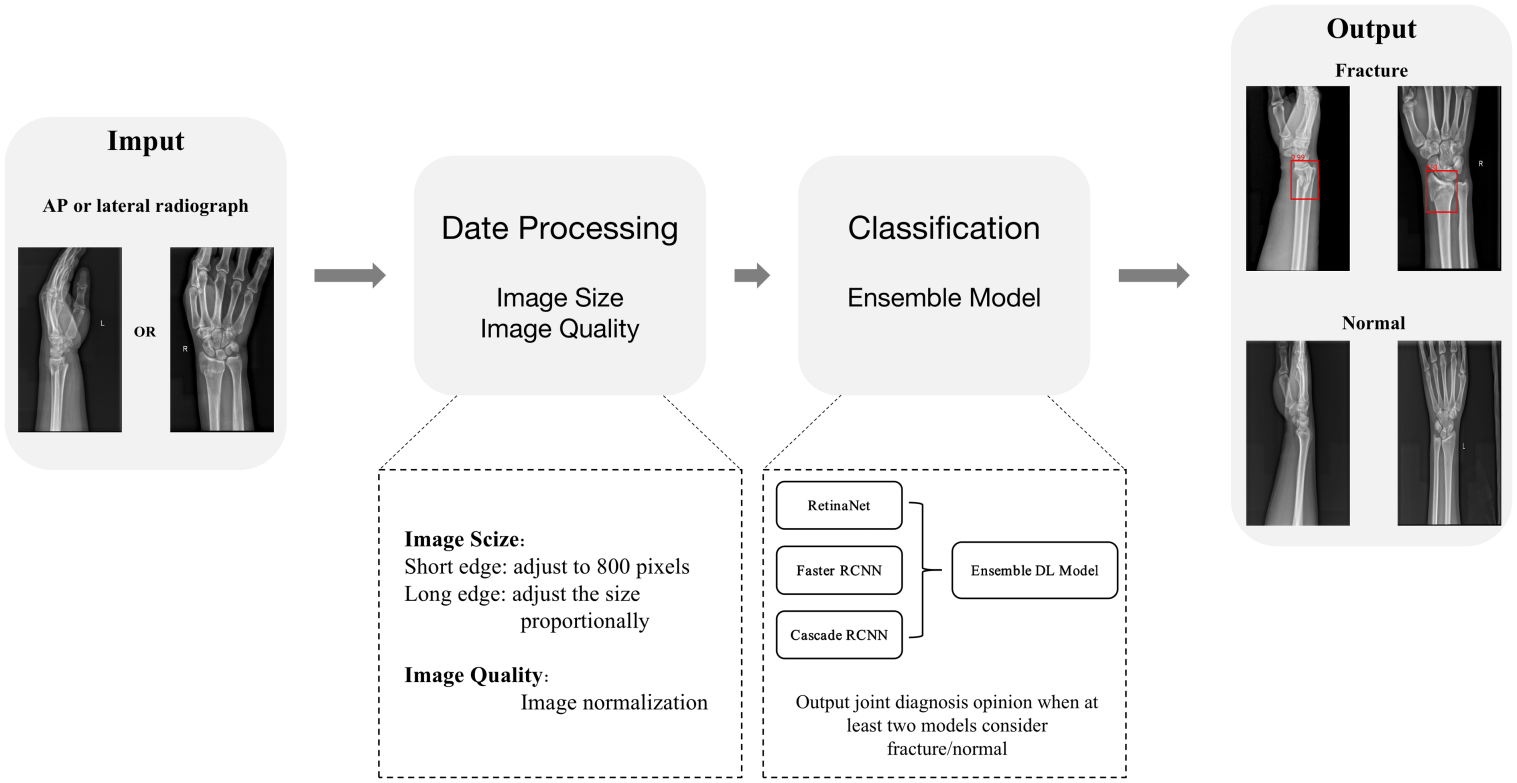
Deep learning ensemble model structure diagram.

Based on the three pre-trained models, we developed an ensemble model combining multiple deep learning algorithms to judge whether the input X-ray image of the distal radius is fractured or not. When at least two models considered fracture/normal, a joint diagnosis opinion was produced, and the average probability of fracture was calculated (Figure 4).

We used the Ubuntu 16.04 operating system^2^ to run the PyTorch deep learning framework in an environment equipped with NVIDIA V100 GPU (CUDA version 10.2, cuDNN version 7.6.5),^3^ and 32 GB of Video Random Access Memory (VRAM).

### Evaluation of deep learning performance

**Figure 5:**
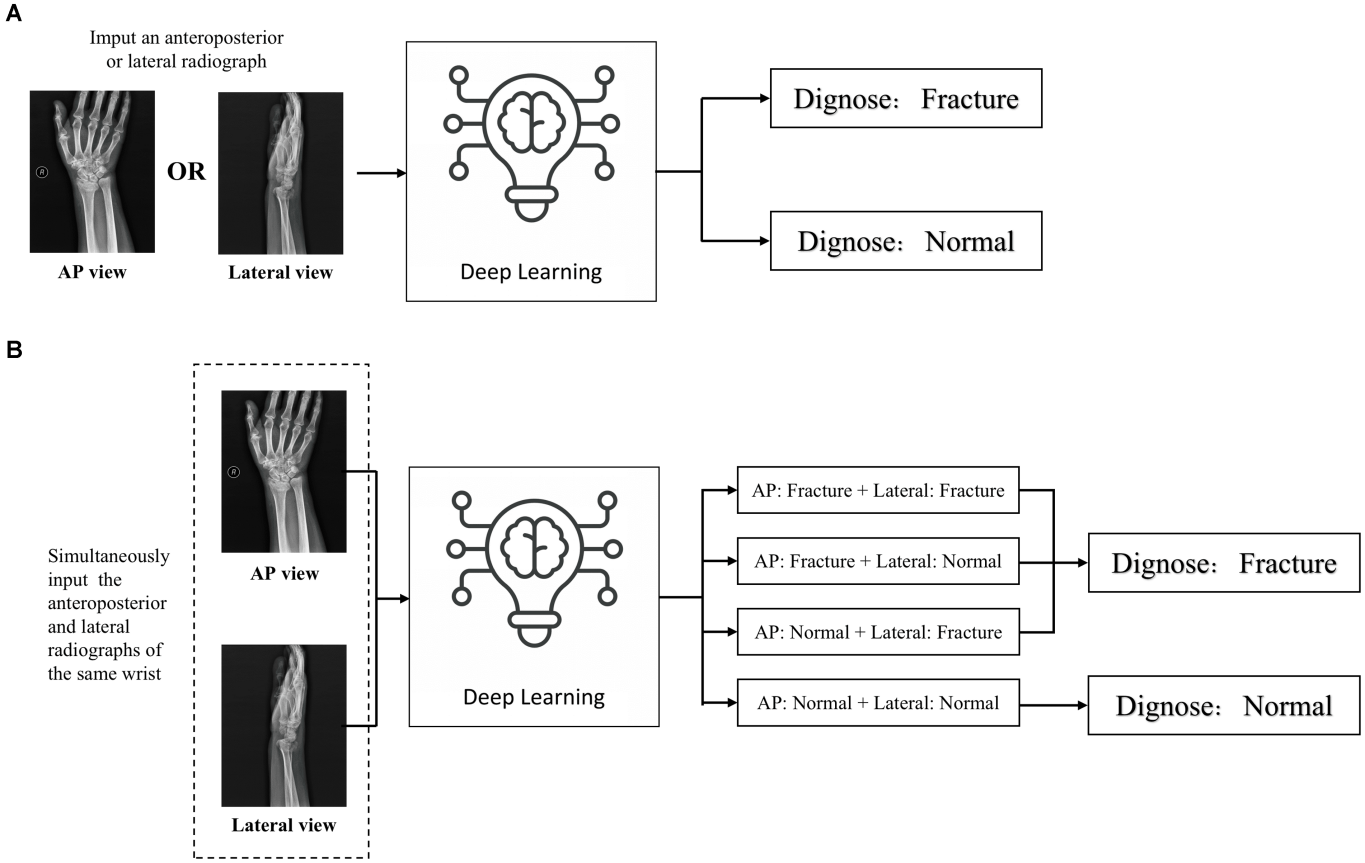
Two detection modes of deep learning detection model **(A)** When inputting a single front view or lateral X-ray images, judge whether there is a fracture in the distal radius region according to a single radiograph; **(B)** When the AP and lateral X-ray images of the ipsilateral wrist joint of a patient are inputted at the same time, the comprehensive diagnosis concerning the distal radius region is made based on the diagnostic results of the two images. If any one of the anteroposterior or lateral images is diagnosed as fractured, the wrist joint is diagnosed as DRF; if no fractures are detected in both the anteroposterior and lateral images, the distal radius region is judged as unfractured.

An independent test set was used to test the performance of trained deep learning models and evaluate its ability to recognize fractures in X-ray images. Evaluated the diagnostic performance of the models in three types of radiographic images: AP + lateral view, AP view, and lateral view (Figure 5A), and assess the final clinical diagnosis results for the wrist joint unit (each wrist joint has one AP image and one lateral image) (Figure 5B). Different score probability thresholds were set for the trained deep learning model to draw the Receiver Operating Characteristic (ROC) curve, and the area under the curve (AUC) was calculated to evaluate the performance of the model. The optimal diagnostic score threshold of the model is set on the corresponding score threshold when Youden’s index reaches the maximum value. According to the optimal score threshold, the accuracy, sensitivity and specificity of the model were calculated to evaluate the performance of the deep learning models. Finally, the accuracy, sensitivity, and specificity of the ensemble model were calculated. To obtain point and interval estimators, we used the bootstrap method to resample test date with 1,000 times on the test dateset; the mean accuracy, sensitivity, specificity, and 95% CI were computed.

### Assessment of diagnostic performance by medical personnel

To evaluate the diagnostic performance of medical personnel, we established an orthopedic diagnosis team consisting of three attending orthopedists and a radiology diagnosis team consisting of three attending radiologists. All the included orthopedic attending physicians have at least 3 years of experience in trauma orthopedics and possess professional X-ray image reading skills. Attending radiologists included had at least 3 years of professional experience in radiological diagnosis. None of the above physicians participated in data collection and labeling.

Participating physicians were informed to perform independent analysis and diagnosis of the data in the test set. Diagnostic tests were performed by shuffling the test set data using a randomization procedure.^4^ To ensure consistent conditions between the deep learning model and the physician, the physician was not informed of the injury mechanism and patient age during the entire process of test. In addition, in order to avoid any intra-group or inter-group influence, all participants were not informed the research plan and diagnostic tests were conducted separately.

Finally, we calculated the average accuracy, sensitivity and specificity of each group of physicians in diagnosis and compared their performance with that of the deep learning model.

### Statistical analyses

Continuous variables were presented as median [interquartile range (IQR)]. Categorical variables were expressed by counts and percentages. For the comparison of baseline characteristics among different datasets, the ANOVA test was used for continuous variables and *χ*^2^ test was used for categorical variables. Accuracy, sensitivity, and specificity were selected as diagnostic performances, and the corresponding 95% confidence intervals were estimated using bootstrapping with 1,000 bootstraps. The ANOVA test was used to compare diagnostic performances of ensemble model, orthopedists and radiologists. The bootstrapping was performed using packages “boot” of R 4.1.2 (The R Foundation for Statistical Computing, Vienna, Austria). Other statistical analyses were performed using SAS Statistics software (version 9.4, SAS Institute Inc., Cary, North Carolina, United States). All statistical tests were two-sided, and *p* < 0.05 was considered statistically significant.

## Results

### Demographic data of patients

The age difference between the fractured and non-fractured groups was not statistically significant (*p* = 0.433), while there is a statistically significant gender difference between the two groups (*p* < 0.001). In addition, there were no significant differences in age (*p* = 0.619) or gender (*p* = 0.817) among the training set, validation set, and test set. Detailed statistical analysis results are provided in Tables 1, 2.

**Table 1:** Clinical information of included patients (Diagnosis-based classification).

Variables	Patients with DRF (*n* = 1,620)	Patients without DRF (*n* = 1,620)	Total (*n* = 3,240)	*p* value
Age, median (IQR)	59 (51–67)	60 (51–69)	60 (51–67)	0.433
Sex, *n* (%)				
Male	494 (30.49)	841 (51.91)	1,335 (41.20)	<0.001
Female	1,126 (69.51)	779 (48.09)	1905 (58.80)	

IQR, interquartile range.

**Table 2:** Clinical information of included patients (Divide according to the dataset).

Variables	Training set (*n* = 2,268)	Validation set (*n* = 486)	Testing set (*n* = 486)	Total (*n* = 3,240)	*p* value
Age, median (IQR)	60 (51–67)	61 (51–68)	59 (51–68)	60 (51–67)	0.619
Sex, *n* (%)					
Male	939 (41.40)	201 (41.36)	195 (40.12)	1,335 (41.20)	0.871
Female	1,329 (58.60)	285 (58.64)	291 (59.88)	1905 (58.80)	

IQR, interquartile range.

### Performance of the deep learning models

**Figure 6:**
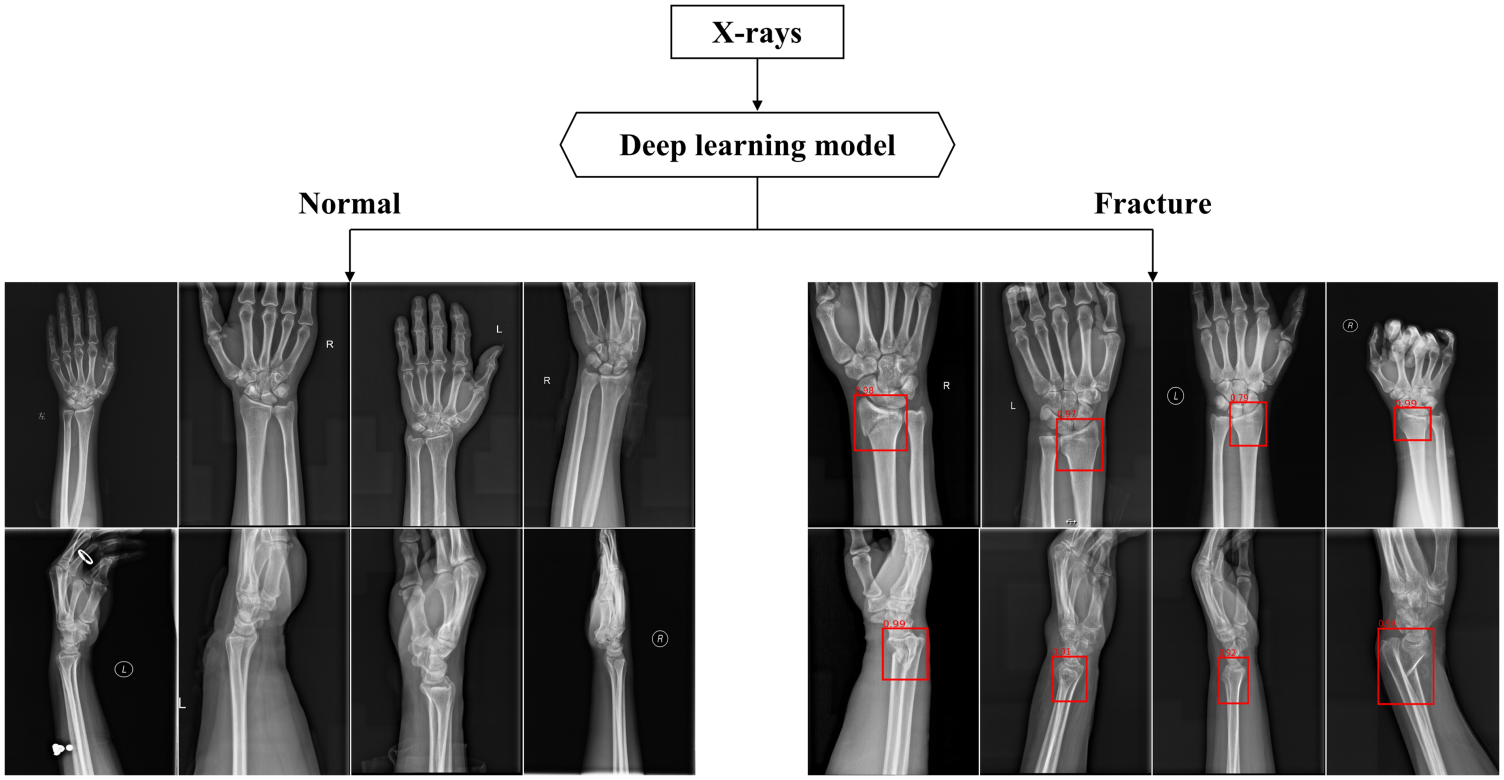
Part of output X-rays dignose results. The above figures show some of the output results in the test dataset. The algorithm used red rectangles to mark suspicious fractures and provided corresponding fracture prediction probability values.

After training, the algorithm is able to use the previously learned features to diagnose images in the test database. If the diagnosis result is DRF, a red rectangle will be displayed on the suspicious area and the predicted probability value will also appear (as shown in Figure 6).

**Figure 7:**
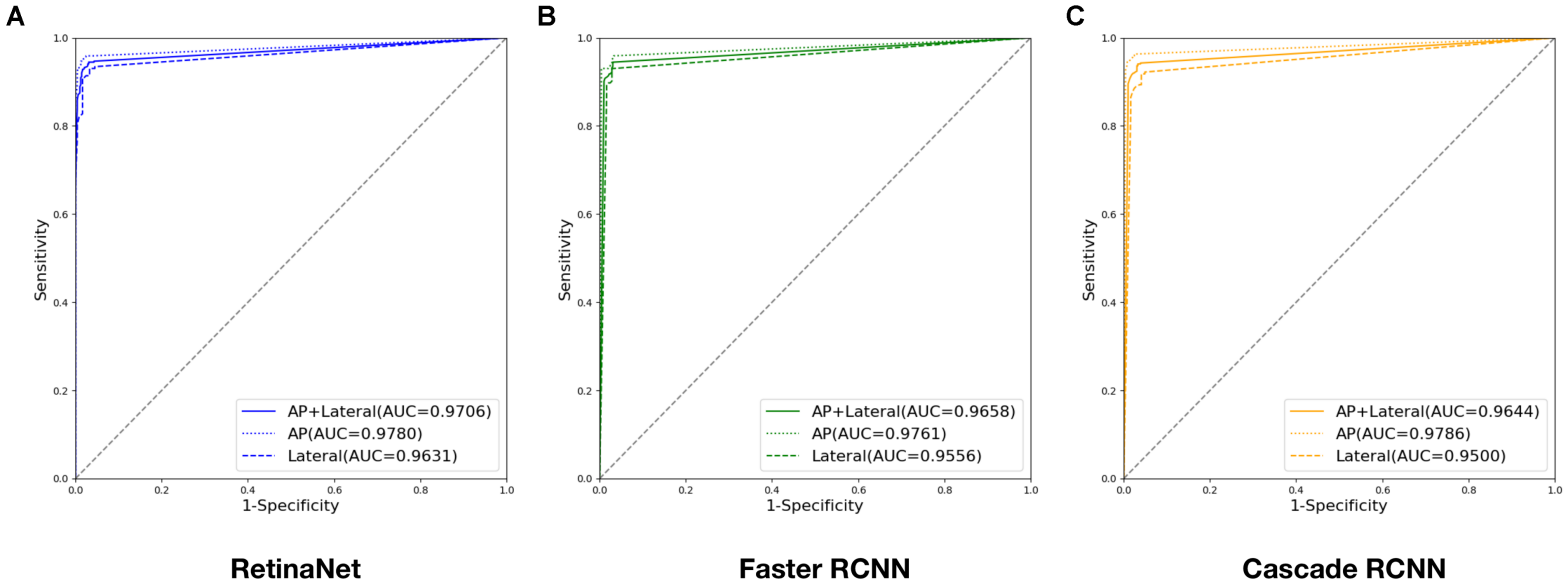
ROC curves output by three deep models during testing: **(A)** ROC curve output by RetinaNet; **(B)** ROC curve output by Faster RCNN; **(C)** ROC curve output by Cascade RCNN. (Four digits after the decimal point are kept for this experiment to ensure data precision).

Evaluated the deep learning diagnostic models with the test set, and the ROC curves of RetinaNet, Faster RCNN, and Cascade RCNN are shown in Figure 7. The AUC of RetinaNet for diagnosing fractures on the test set is 0.9706, with an AUC of 0.9780 for AP images and an AUC of 0.9631 for lateral images. The AUC of Faster RCNN for diagnosing fractures on the test set is 0.9658, with an AUC of 0.9761 for AP images and an AUC of 0.9556 for lateral images. The AUC of Cascade RCNN for diagnosing fractures on the test set is 0.9644, with an AUC of 0.9786 for AP images and an AUC of 0.9500 for lateral images.

When the maximum value of Youden’s index is 91.41%, the corresponding optimal score threshold for RetinaNet is 0.71. When the maximum value of Youden’s index is 91.41%, the corresponding optimal score threshold for Faster RCNN is 0.65. When the maximum value of Youden’s index is 90.79%, the corresponding optimal score threshold for Cascade RCNN is 0.66. The detailed results of accuracy, sensitivity, and specificity diagnosed by three deep learning models are shown in Table 3.

**Table 3:** Diagnostic performance of each deep learning model.

Model	Type	Date type	Accuracy % (CI)	Sensitivity % (CI)	Specificity % (CI)
RetinaNet	One-stage	Testing set (AP + Lateral)	95.71 (94.17–96.83)	94.47 (92.05–96.11)	96.94 (94.82–98.16)
		AP	96.52 (94.27–97.75)	95.90 (92.21–97.54)	97.14 (93.88–98.37)
		Lateral	94.89 (92.23–96.32)	93.03 (89.34–95.90)	96.73 (93.06–98.37)
		Wrist	95.71 (93.46–97.34)	97.54 (94.67–98.77)	94.29 (90.20–96.33)
Faster RCNN	Two-stage	Testing set (AP + Lateral)	95.71 (94.22–96.83)	94.47 (92.05–96.11)	96.94 (94.90–98.16)
		AP	96.32 (94.07–97.55)	95.9 (91.80–97.54)	96.73 (93.05–97.96)
		Lateral	95.09 (92.64–96.52)	93.03 (89.34–95.90)	97.14 (93.47–98.37)
		Wrist	95.91 (93.46–97.30)	97.13 (93.21–98.36)	94.29 (90.42–96.33)
Cascade RCNN	Multi-stage	Testing set (AP + Lateral)	95.4 (93.66–96.42)	94.06 (91.39–95.70)	96.73 (94.49–97.96)
		AP	96.93 (94.89–98.16)	96.31 (93.03–97.95)	97.55 (93.82–98.78)
		Lateral	93.87 (91.00–95.50)	91.8 (86.48–94.26)	95.92 (92.65–97.55)
		Wrist	95.5 (93.05–96.73)	97.54 (93.60–98.77)	93.47 (89.39–95.92)
Ensemble model	–	Testing set (AP + Lateral)	97.03 (95.71–97.96)	95.7 (93.44–97.13)	98.37 (96.73–99.18)
		AP	97.75 (96.11–98.77)	97.13 (93.75–98.36)	98.37 (95.10–99.18)
		Lateral	96.32 (93.87–97.55)	94.26 (90.57–96.31)	98.37 (95.10–99.18)
		Wrist	97.55 (95.71–98.57)	98.36 (95.90–99.59)	96.73 (93.47–98.37)

**Figure 8:**
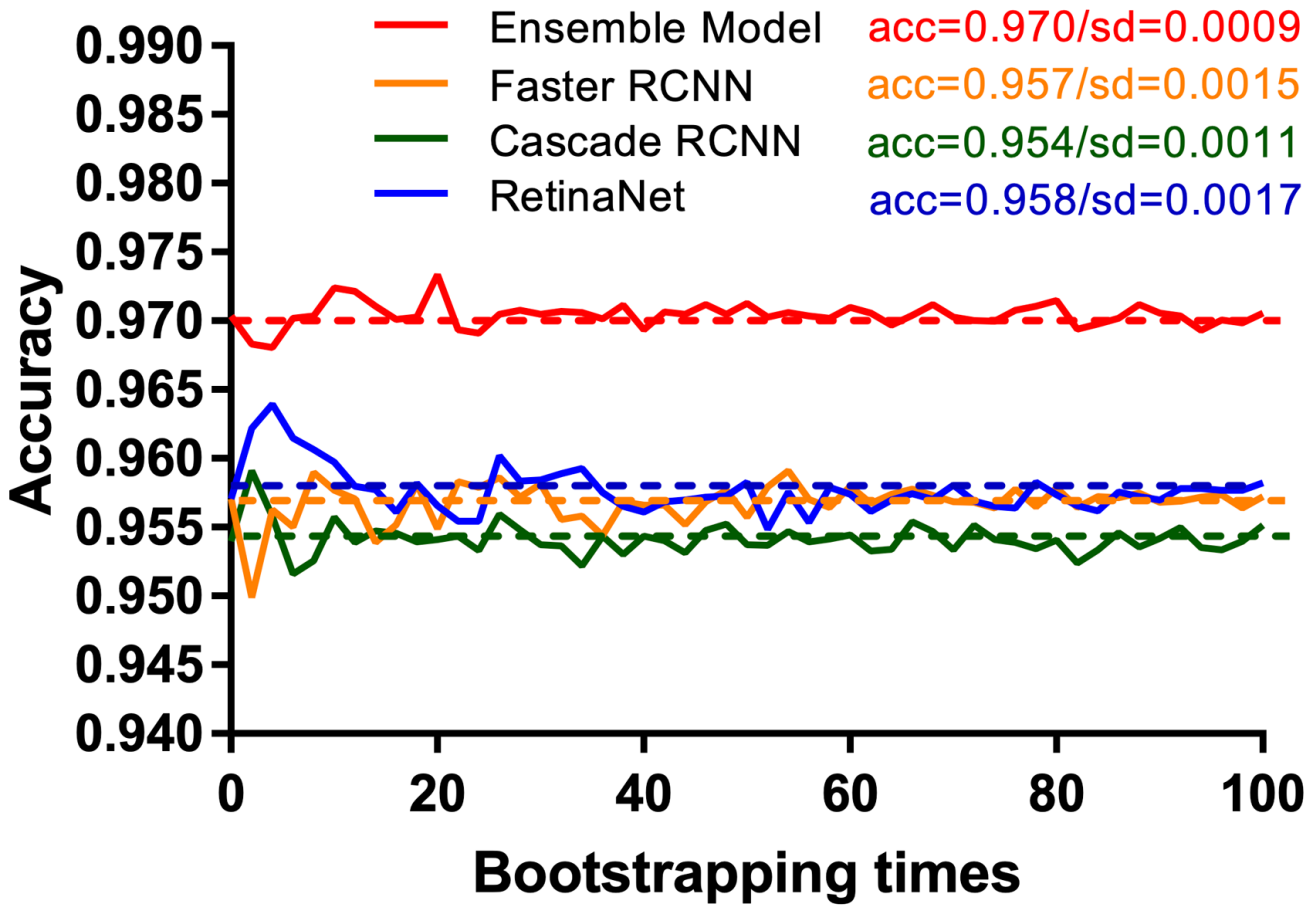
Under the test set, our ensemble model (red line) shows higher accuracy and stability than RetinaNet (blue line), Faster RCNN (orange line), and Cascade RCNN (green line). This study uses the bootstrap method. The X-axis and Y-axis, respectively, represent the number of times and accuracy of each resampling.

**Figure 9:**
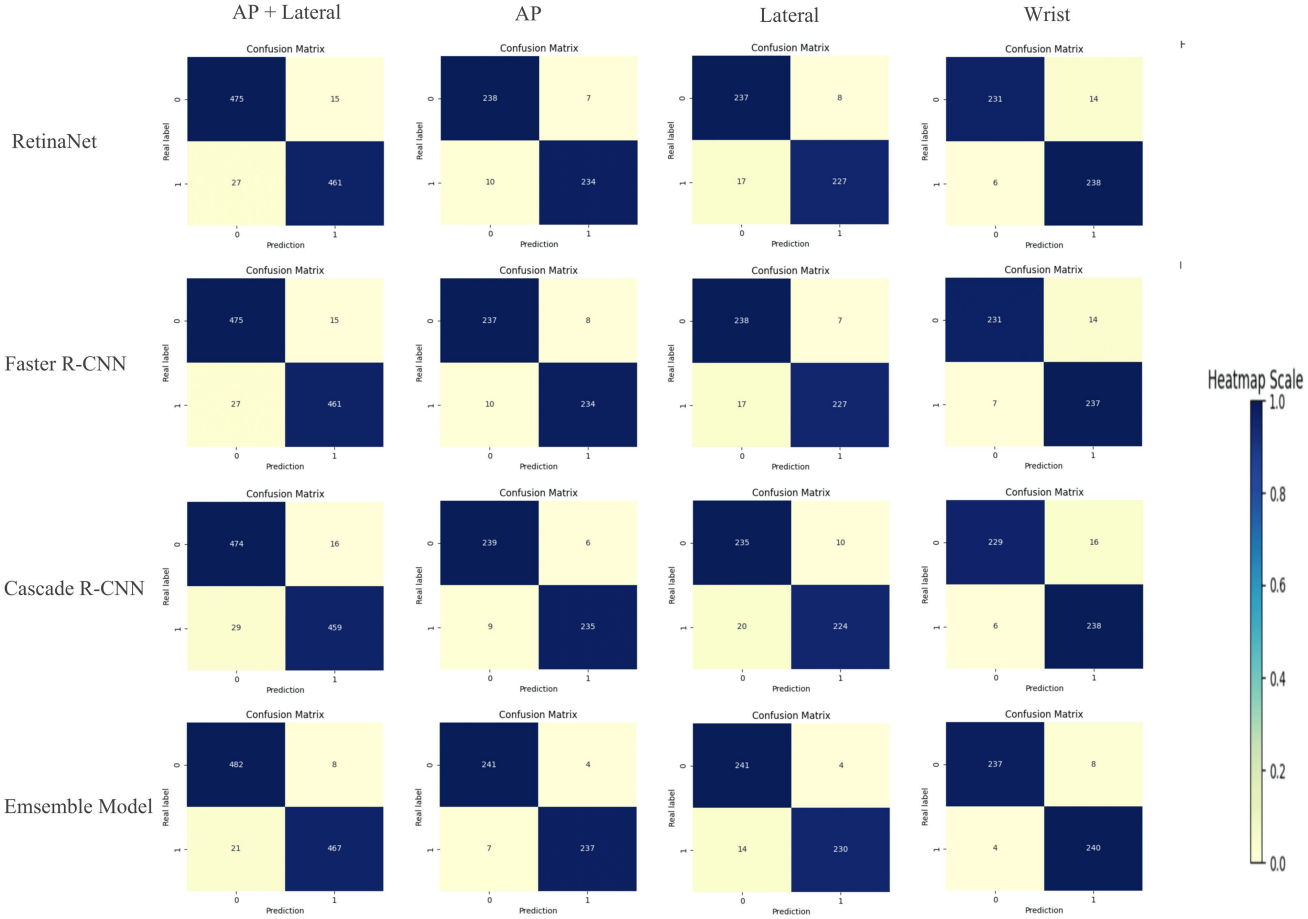
Confusion matrix.

Compared with RetinaNet, Faster RCNN, and CASCADE RCNN, the ensemble model performed better on the test set, with an accuracy of 97.03% (95.71–97.96%), a sensitivity of 95.70% (93.44–97.13%) and a specificity of 98.37% (96.73–99.18%). The ensemble model outperformed the individual models with higher accuracy and lower standard deviation (Figure 8). When the diagnostic performance of DRFs were counted in units of wrist joints, the accuracy, sensitivity, and specificity reached 97.55% (95.71–98.57%), 98.36% (95.90–99.59%), and 96.73% (93.47–98.37%), respectively, all of which were superior to a single model (Table 3). The detailed results can be seen in the confusion matrix of Figure 9. We therefore use this deep learning ensemble model for DRFs detection.

### Performance of the medical personnel

The average accuracy, sensitivity, and specificity of the orthopedic attending physician group were 93.69% (91.89–94.99%), 91.94% (89.21–93.92%), and 95.44% (93.06–97.00%) respectively. The average accuracy, sensitivity, and specificity of the attending radiologist group were 92.53% (90.73–94.06%), 90.44% (87.43–92.55%), and 94.62% (92.24–96.33%) respectively. The data is shown in Table 4. Detailed diagnoses results of each physician are provided in the Supplementary materials.

**Table 4:** Diagnostic performance of medical personnel.

	Date type	Accuracy % (CI)	Sensitivity % (CI)	Specificity % (CI)
Orthopedist group (average)	Testing set (AP + Lateral)	93.69 (91.89–94.99)	91.94 (89.21–93.92)	95.44 (93.06–97.00)
	AP	94.48 (91.96–96.18)	93.30 (89.89–96.27)	95.65 (92.05–97.82)
	Lateral	92.91 (90.02–94.82)	90.57 (86.61–94.13)	95.23 (91.59–97.55)
	Wrist	93.87 (91.29–95.64)	95.90 (92.21–97.54)	91.84 (87.48–95.03)
Radiologist group (average)	Testing set (AP + Lateral)	92.53 (90.73–94.06)	90.44 (87.43–92.55)	94.62 (92.24–96.33)
	AP	93.32 (90.72–95.29)	91.66 (87.70–94.54)	94.96 (91.18–97.01)
	Lateral	91.75 (88.91–93.93)	89.20 (84.97–92.76)	94.29 (90.42–96.33)
	Wrist	92.91 (90.41–94.96)	95.35 (91.39–97.40)	90.48 (86.39–93.47)

CI = 95% confidence interval.

### Comparison of deep learning models and clinical physicians

The results of the ensemble model were compared with those of the clinician group. The results are shown in Table 5.

**Table 5:** Comparison between the ensemble model and physicians.

Factor	Date Type	Ensemble model	Orthopedists	Radiologists	*p*
Accuracy % (CI)	Testing set (AP + Lateral)	97.03 (95.71–97.96)	93.69 (91.89–94.99)	92.53 (90.73–94.06)	<0.001
	AP	97.75 (96.11–98.77)	94.48 (91.96–96.18)	93.32 (90.72–95.29)	<0.001
	Lateral	96.32 (93.87–97.55)	92.91 (90.02–94.82)	91.75 (88.91–93.93)	<0.001
	Wrist	97.55 (95.71–98.57)	93.87 (91.29–95.64)	92.91 (90.41–94.96)	<0.001
Sensitivity % (CI)	Testing Set (AP + Lateral)	95.7 (93.44–97.13)	91.94 (89.21–93.92)	90.44 (87.43–92.55)	<0.001
	AP	97.13 (93.75–98.36)	93.30 (89.89–96.27)	91.66 (87.70–94.54)	<0.001
	Lateral	94.26 (90.57–96.31)	90.57 (86.61–94.13)	89.20 (84.97–92.76)	<0.001
	Wrist	98.36 (95.90–99.59)	95.90 (92.21–97.54)	95.35 (91.39–97.40)	<0.001
Specificity % (CI)	Testing set (AP + Lateral)	98.37 (96.73–99.18)	95.44 (93.06–97.00)	94.62 (92.24–96.33)	<0.001
	AP	98.37 (95.10–99.18)	95.65 (92.05–97.82)	94.96 (91.18–97.01)	<0.001
	Lateral	98.37 (95.10–99.18)	95.23 (91.59–97.55)	94.29 (90.42–96.33)	<0.001
	Wrist	96.73 (93.47–98.37)	91.84 (87.48–95.03)	90.48 (86.39–93.47)	<0.001

The ensemble model outperformed the orthopedic and radiologist groups in terms of diagnostic accuracy, sensitivity, and specificity. When collecting statistics on the wrist joint, the ensemble model still outperformed the performance of the orthopedic surgeon group and the radiologist group in terms of diagnostic accuracy, sensitivity, and specificity.

## Discussion

The wrist joint is one of the most important joints in the body, with high frequency of movement, and a relatively high requirement for functional recovery if injured (2). The misdiagnosis or delayed treatment of DRFs can cause traumatic arthritis of the wrist joint, which can seriously affect the function of the hand. Especially for elderly people, the recovery after a bone fracture is relatively slow. If not diagnosed or treated in a timely manner, it may lead to adverse consequences such as weakness, deformity, shortening, stiffness, pain, and limited mobility of the wrist joint, thereby affecting the quality of daily life (3, 8). It can also have a certain negative impact on mental health, which may increase the psychological burden and anxiety of elderly patients, such as anxiety, depression, and loss of independence (4). Therefore, timely and accurate post-fracture diagnosis is crucial to the treatment and rehabilitation.

In this study, we constructed an ensemble model consisting of three different deep learning algorithms for the detection of DRFs. Our research confirmed that the trained and integrated model demonstrates excellent performance in distinguishing fractured or unfractured in the structure of the distal radius. The overall dignostic accuracy of the model has reached 97.03%, the sensitivity 95.70%, the specificity 98.37%. These results were better than the performance of orthopedic attending physicians and radiology attending physicians. In the diagnostic analysis of subdivided AP or lateral radiographs, it is also significantly better than the attending physicians in orthopedics and radiology. When using the wrist joint as a unit and simultaneously inputting two X-ray images of the AP and lateral positions for comprehensive diagnosis, the accuracy rate can reach 97.75%, with sensitivity and specificity of 98.36 and 96.73%, respectively, which is better than that of physicians in orthopedics and radiology.

Wrist X-ray examination in the AP and lateral views are the most commonly used imaging examination for diagnosing DRFs. However, the misdiagnosis of fractures in radiology is a common problem for non-specialist physicians or radiology resident doctors, especially in emergency environments, which can easily lead to extra harm or delayed treatment for patients (27). According to relevant studies, misdiagnosis of fractures accounts for 24% of harmful diagnostic errors in the emergency department, and misdiagnosis of hand and wrist fractures accounts for 29% of all misdiagnosed fractures (28). In addition, for patient admissions during night shifts, inconsistent imaging diagnosis opinions of fractures are more common, which may be related to non-expert reading and fatigue (29).

Deep learning is a branch of artificial intelligence that trains models by inputting data such as images, text, or sound, and enables models to learn to perform more complex classification tasks (30). Compared to traditional machine learning methods, deep learning has higher performance. In the field of medical image analysis, trained deep learning algorithms can simulate clinical doctors’ judgments and accurately detect fractures (31). Deep learning algorithms for fracture detection offer significant advantages in clinical settings. Firstly, AI can be an effective tool for triage in emergency situations. AI can perform preliminary screening and discover positive results, which can allow doctors to prioritize the imaging data with fracture signs to reduce adverse effects caused by delayed diagnosis. Secondly, AI-assisted fracture identification can enhance the diagnostic ability of both clinical and radiological doctors. It can detect small lesions that are easily overlooked by human eyes, and also reduce the possibility of decreased attention and fracture detection ability due to visual fatigue and mental exhaustion caused by long shifts. Lastly, another potential benefit of AI is shortening reading and diagnosis time. For radiologists who need to review a large number of images every day and emergency doctors who need to make multiple urgent judgments, AI is an important auxiliary tool because it can save a lot of time. In short, the application of AI-assisted fracture diagnosis models can reduce missed diagnoses of DRFs. It can quickly and effectively diagnose patient imaging data, thereby speeding up medical workflows and improving patient outcomes.

Previous researches investigated the feasibility of using deep learning to detect fractures on X-ray films and showed good results, which is consistent with our study (Table 6). Zech et al. (17) trained the Faster RCNN model for the detection of carpal fractures in children groups, and reached an accuracy of 88%, a sensitivity of 88%, and a specificity of 89%, and the use of this model was effective in improving the diagnostic ability of radiology trainees. Ashkani-Esfahani et al. (32) trained two deep learning models, Inception V3 and Renet-50, for the detection of ankle fractures. The results showed that Inception V3 had better diagnostic performance, with a sensitivity of 98.7% and a specificity of 98.6%. Yoon et al. (33) trained a DCNN model based on the EfficientNetB3 structure to classify normal and scaphoids scaphoids with a sensitivity and specificity of 87.1 and 92.1%, respectively. Liu et al. (34) applied a Faster RCNN algorithm for training and FIF detection on X-ray images and compared it with orthopedic attending. The results showed that the Faster RCNN algorithm performed better in terms of accuracy, specificity and efficiency. Li et al. (35) constructed an artificial intelligence deep learning model framework including target detection, data preprocessing of radiographs and classification for detecting vertebral fractures. The AI model of this integrated approach had excellent accuracy (93%), sensitivity (91%), and specificity (93%) in detecting lumbar vertebral fractures. In addition to the diagnosis of fractures, AI has also improved the determination of bone age, as well as the diagnosis of other orthopedic diseases such as osteoarthritis, spondylolisthesis, and bone tumors (18–20, 36).

**Table 6:** Summary of the performance of DL in fracture diagnosis.

Fracture diagnosis	Data size	Accuracy	Sensitivity	Specificity	Reference
Pediatric wrist fractures	395	0.88	0.88	0.89	Zech et al. (17)
Ankle fractures	2,100	0.99	0.99	0.99	Ashkani-Esfahani et al. (32)
Scaphoid fractures	11,838	0.91	0.88	0.92	Yoon et al. (33)
Femoral intertrochanteric fractures	700	0.88	0.89	0.87	Liu et al. (34)
Vertebral fractures	941	0.93	0.91	0.93	Li et al. (35)
Distal radius fractures (this study)	6,536	0.97	0.96	0.98	-

However, we must be aware that algorithms still inevitably produce diagnostic errors and potential medical risks. Therefore, it is currently best to use AI as a second expert to assist clinicians in making a diagnosis, rather than replacing doctors for the final diagnosis.

Our study also has some limitations. (1) This is a retrospective study, and all imaging examinations did not have complete clinical medical records. During the testing process, the participating doctors diagnosed only through imaging data, just like deep learning models. However, in a real clinical environment, non-radiologists can examine patients physically and obtain detailed medical history information, while radiologists can also access patient medical records to identify areas of concern, which, combined this with X-ray films, improves the sensitivity and specificity of fracture diagnosis. Therefore, the results of the physician group in this study only represent the level achieved when diagnosing based on imaging data merely, and cannot represent the diagnostic level in a completely real clinical environment. (2) The dataset in the database only contains data from a single medical center. Although the dataset is large and experimental results demonstrate excellent performance of the model, obtaining more images from different medical institutions would increase the diversity of data sources, which may further improve the reliability of the results. (3) The ensemble model we trained can be used for detecting DRFs, but cannot further classify the type of fractures. Accurately determining the type of fracture is also important for treatment as different types require different treatment plans. In the future, we will further develop related models to achieve intelligent classification of DRFs and provide assistance in determining more accurate treatment plans.

Although these factors may affect the performance of our detection model, but our research results are still worth serious consideration. This fast, accurate, and intelligent fracture detection algorithm can be used by junior doctors in emergency rooms and outpatient clinics to assist clinical diagnosis. This not only helps reduce clinical workloads, but also the risk of misdiagnosis.

## Conclusion

We have developed an ensemble model based on deep learning algorithms for detecting DRFs and demonstrated excellent diagnostic performance. The results of this study demonstrate the feasibility of the fracture detection technology based on deep learning, and will contribute to further research on fracture detection in more types and locations in the future. At the same time, we will build larger datasets in the future and train advanced algorithms to achieve automatic detection of DFR and intelligent determination of fracture types. In summary, this fast and accurate diagnostic tool is expected to become the second expert for doctors in clinical practice, improving the accuracy of diagnosis of DRFs and reducing their burden in clinical work.

## Data availability statement

The original contributions presented in the study are included in the article/Supplementary material, further inquiries can be directed to the corresponding authors.

## Ethics statement

This study was performed in accordance with the declaration of Helsinki and approved by the Medical Science Research Ethics Committee. All researches were performed in accordance with relevant guidelines and regulations. Written informed consent was obtained from the individual(s) for the publication of any potentially identifiable images or data included in this article.

## Author contributions

JZ and ZY contributed to the conception and design of the study. ZL, TH, MiX, and HL contributed to the acquisition and processing data. YX, HZ, and JY assisted in the conducting experiment. SL and MaX performed the statistical analysis. JZ, ZL, and HL wrote the draft of the manuscript. HW and YF wrote the sections of the manuscript. ZY, PL, and LL contributed to the manuscript revision and supervision. All authors contributed to the article and approved the submitted version.

## Funding

This work was supported by the National Natural Science Foundation of China (No. 81974355 and No. 82172524), the Artificial Intelligence Major Special Project of Hubei (NO. 2021BEA161) and National Innovation Platform Development Program (No. 2020021105012440).

## Conflict of interest

The authors declare that the research was conducted in the absence of any commercial or financial relationships that could be construed as a potential conflict of interest.

## Publisher’s note

All claims expressed in this article are solely those of the authors and do not necessarily represent those of their affiliated organizations, or those of the publisher, the editors and the reviewers. Any product that may be evaluated in this article, or claim that may be made by its manufacturer, is not guaranteed or endorsed by the publisher.

## Footnotes

^1^ https://github.com/wkentaro/Labelme

^2^ http://www.ubuntu.com

^3^ http://developer.nvidia.com

^4^ https://www.randomizer.org
