# Supplementary material for: Deep learning assisted diagnosis system: improving the diagnostic accuracy of distal radius fractures
Source: Front Med (Lausanne). 2023 Aug 17;10:1224489. doi: 10.3389/fmed.2023.1224489 (PMC10471443; doi:10.3389/fmed.2023.1224489)
Supplement: Supplementary file 1 [file Table_1.DOCX]

# Supplementary Tables

**Supplementary table 1. Detailed results for the physician group**

| Doctor | Date type | Accuracy % (CI) | Sensitivity % (CI) | Specificity % (CI) |
| --- | --- | --- | --- | --- |
| orthopedist 1 | Testing Set  (AP+Lateral) | 93.66 (91.82-94.89) | 91.80 (88.93-93.85) | 95.51 (93.12-96.94) |
|  | AP | 94.48 (91.82-96.11) | 93.03 (89.34-95.90) | 95.92 (91.84-97.96) |
|  | Lateral | 92.84 (89.18-94.68) | 90.57 (86.07-93.80) | 95.10 (91.02-97.14) |
|  | Wrist | 93.87 (91.00-95.50) | 95.90 (92.21-97.54) | 91.84 (97.35-94.69) |
| orthopedist 2 | Testing Set  (AP+Lateral) | 93.76 (91.82-95.09) | 92.01 (88.93-93.85) | 95.51 (93.06-96.94) |
|  | AP | 94.68 (92.16-96.32) | 93.85 (90.57-96.72) | 95.51 (91.53-97.14) |
|  | Lateral | 92.84 (90.18-94.89) | 90.16 (85.66-93.03) | 95.51 (91.53-97.14) |
|  | Wrist | 94.07 (91.41-95.71) | 95.90 (92.21-97.54) | 92.24 (87.76-94.69) |
| orthopedist 3 | Testing Set  (AP+Lateral) | 93.66 (91.82-94.89) | 92.01 (88.93-94.06) | 95.31 (92.86-96.73) |
|  | AP | 94.27 (91.62-95.91) | 93.03 (88.93-95.49) | 95.51 (91.84-97.14) |
|  | Lateral | 93.05 (90.18-94.89) | 90.98 (85.66-93.59) | 95.10 (91.16-97.14) |
|  | Wrist | 93.66 (91.00-95.50) | 95.90 (91.80-97.54) | 91.43 (86.94-94.29) |
| radiologist 1 | Testing Set  (AP+Lateral) | 92.94 (90.90-94.38) | 91.19 (88.52-93.44) | 94.69 (91.84-96.33) |
|  | AP | 93.66 (91.00-95.30) | 92.21 (88.11-94.67) | 95.10 (91.02-97.14) |
|  | Lateral | 92.23 (89.57-94.27) | 90.16 (85.66-93.03) | 94.29 (90.20-96.73) |
|  | Wrist | 92.84 (90.18-94.89) | 95.08 (91.39-97.13) | 90.61 (86.42-93.47) |
| radiologist 2 | Testing Set  (AP+Lateral) | 92.43 (90.59-94.17) | 90.16 (87.03-92.42) | 94.69 (91.93-96.33) |
|  | AP | 93.46 (90.59-95.09) | 91.80 (87.30-94.26) | 96.10 (91.43-97.14) |
|  | Lateral | 91.41 (88.55-93.46) | 88.52 (84.02-92.21) | 94.29 (90.39-96.33) |
|  | Wrist | 93.25 (90.59-94.89) | 95.49 (91.39-97.13) | 91.02 (86.53-93.88) |
| radiologist 3 | Testing Set  (AP+Lateral) | 92.23 (90.26-93.76) | 89.96 (86.96-92.21) | 94.49 (91.84-96.12) |
|  | AP | 92.84 (90.09-94.68) | 90.98 (85.73-93.85) | 94.69 (90.20-96.73) |
|  | Lateral | 91.61 (88.75-93.87) | 88.93 (83.26-82.21) | 94.28 (90.20-96.33) |
|  | Wrist | 92.64 (89.78-94.69) | 95.49 (91.70-97.54) | 89.80 (84.90-93.06) |

CI = 95% confidence interval.
